# Uterine Carcinosarcoma in a Young Female: Case Report and Literature Review

**DOI:** 10.7759/cureus.12642

**Published:** 2021-01-11

**Authors:** Noha N Soror, Daniel Woredekal, Lori Hemrock, Gary Gibson, Robert Bennett

**Affiliations:** 1 Internal Medicine, Western Reserve Health Education/Neomed, Warren, USA; 2 Department of Medicine, Trumbull Regional Medical Center, Youngstown, USA; 3 Medical Oncology, The Hope Center for Cancer Care, Warren, USA; 4 Internal Medicine, Steward Healthcare, Warren, USA; 5 Pathology, Steward Health Care, Warren, USA

**Keywords:** iron deficiency anemia (ida), uterine carcinosarcoma, uterine malignancy, uterine adenocarcinoma, uterine cancer

## Abstract

Uterine carcinosarcoma (USC) is a rare, aggressive primary neoplasm of the Uterus that represents less than 5% of all uterine malignancies. It usually affects older females in their seventh decade. USC disproportionally affects black women more and it has a worse prognosis than other endometrial carcinomas.^ ^We present a case of uterine carcinosarcoma in a young Caucasian female who presented with vague symptoms of nausea, vomiting, and severe iron deficiency anemia.

## Introduction

Uterine carcinosarcoma (USC) is a rare, aggressive neoplasm with onset typically in the seventh decade of life. It accounts for less than 5% of all uterine neoplasms. The reported annual incidence of USC is less than two per 100,000. Carcinosarcomas are unique neoplasms in that these tumors contain both malignant carcinomatous and sarcomatous components [1]. USC disproportionally affects black women. In one study, black women had significantly higher incidence rates as compared to white non‐Hispanic females with a rate ratio of 2.33 [2]. Surgical resection of the tumor with/without adjuvant chemotherapy is the mainstay of treatment. The role of radiation therapy remains to be less clear. Combination therapy, although commonly used, is yet to be studied in depth [3]. We present a case of USC in a young Caucasian female.

## Case presentation

A 28-year-old female with a past medical history of iron deficiency anemia, morbid obesity, depression, and anxiety came to the emergency department with vaginal bleeding of a duration of one month, becoming heavy in the five days prior to presentation. Associated symptoms included dizziness and nausea for three days and fatigue and dyspnea on exertion for several weeks. She denied prior sexual activity. She had never undergone a pelvic examination or pap testing. Menarche was at the age of 10, with 28-day cycles until age 16. Since age 16, she had experienced both secondary amenorrhea up to two months at a time and menorrhagia lasting up to 10 days interspersed with several months or regular cycles.

**Figure 1:**
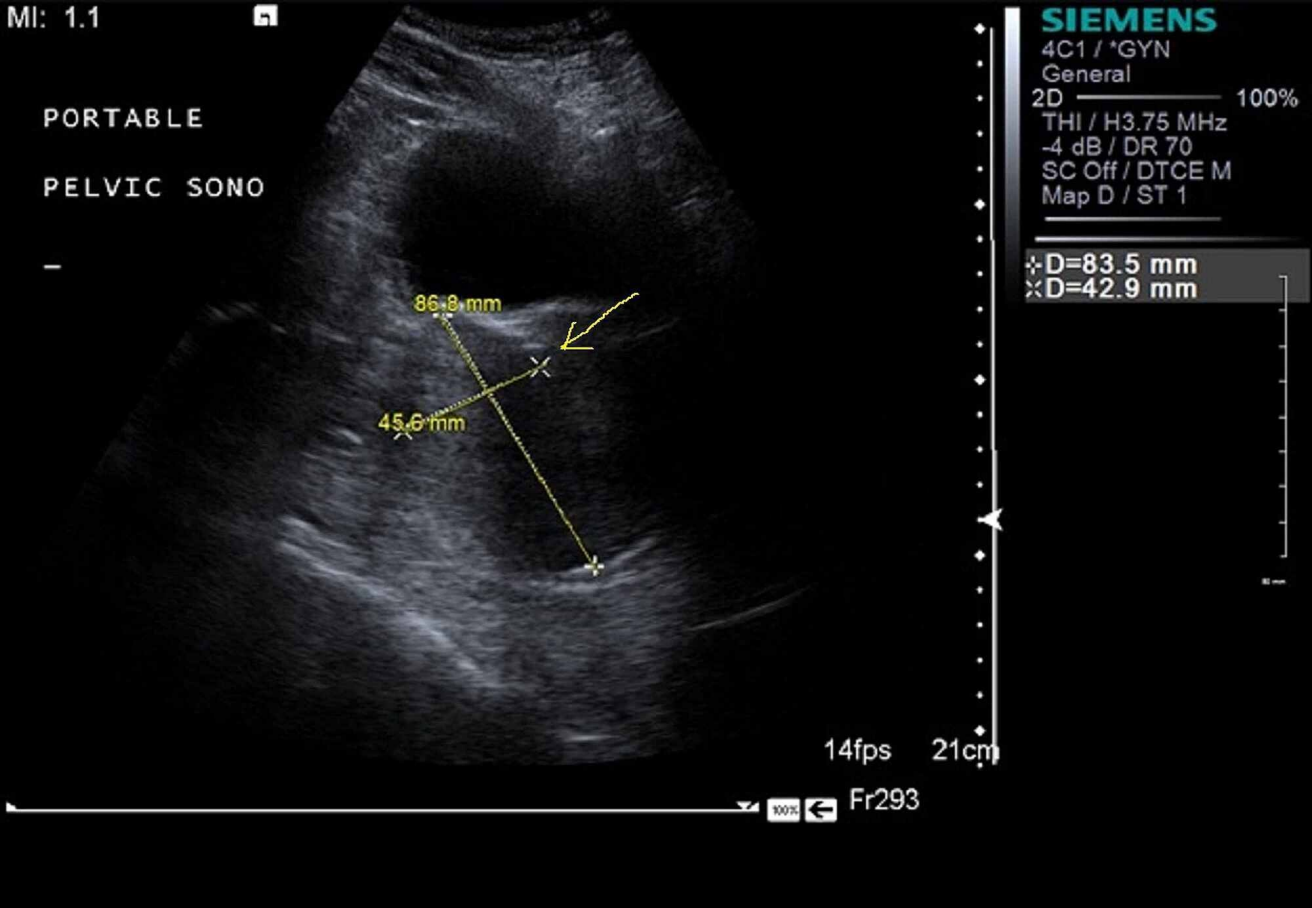
Pelvic ultrasound

Vital signs disclosed tachycardia and a body mass index (BMI) of 61. The examination was notable for morbid obesity and pallor. Labs revealed hemoglobin (Hb) 2.8 gm/dl (12-15), hematocrit (HCT) 9.9 % (35.9-44.6%), mean corpuscular volume (MCV) 57.4 fl (80-96), red cell distribution width (RDW) 22.4% (12.2-16.1), platelets 403 x 103/uL (150-450), and white blood cell (WBC) 7.0 x 103/uL (4.0-11.0). Prothrombin time (PT) and activated partial thromboplastin time (aPTT) were normal. Iron studies confirmed iron deficiency anemia. Cancer antigen (CA) 125 was 63.1 unit/mL (1.5-35). Ultrasound showed an 8x4 cm mass arising from the right ovary as shown in Figure 1. CT abdomen and pelvis showed a large pelvic mass with peripheral calcification measuring up to 8.8 cm, imaging was limited due to the patient's body habitus with streak artifact caused by the patient's skin touching the gantry. Computed tomography (CT) chest was unobtainable for the same reason. Lung bases were clear. There was no evidence of bone or liver metastasis.

**Figure 2:**
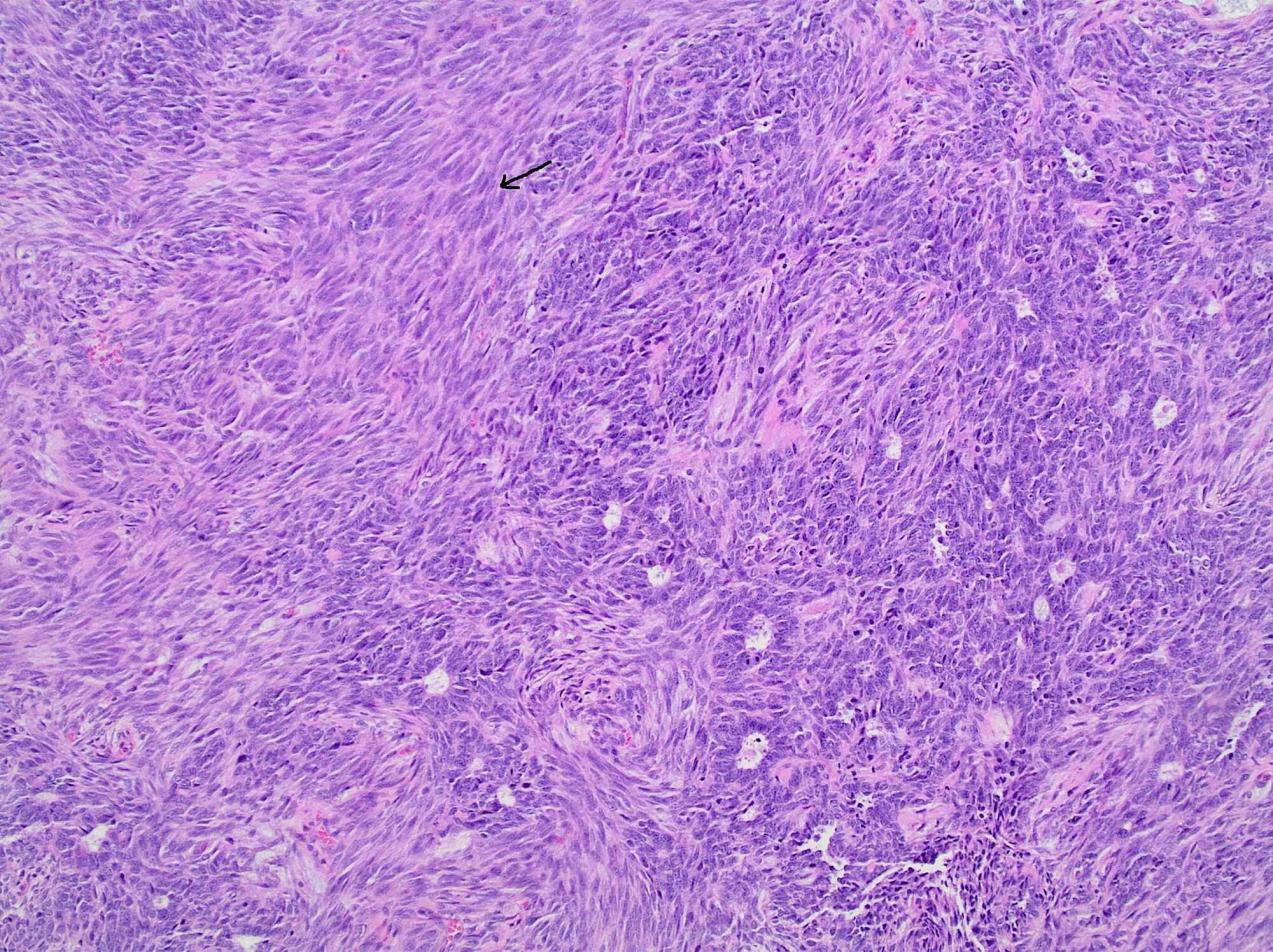
Representative image of glandular and stromal components

**Figure 3:**
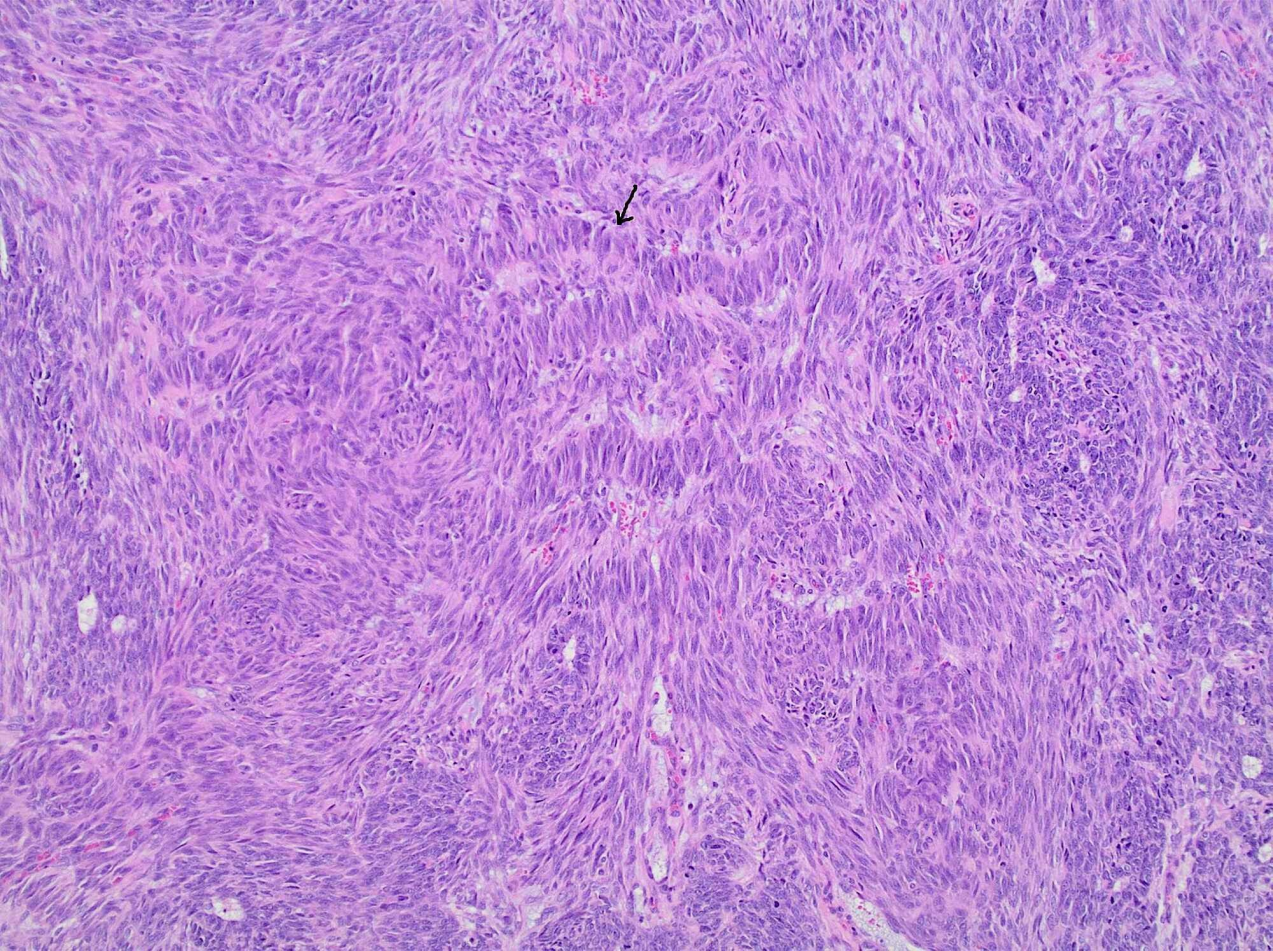
Stromal component with nuclear palisading

A gynecology consultation was obtained. On pelvic examination under generalized anesthesia, she was found to have a friable pelvic mass involving the upper vagina and cervix that bleeds and fragments on touch. The pathology report of a biopsy specimen showed a high-grade uterine carcinosarcoma (Figure 2) with nuclear palisading (Figure 3). The epithelial component consisted of undifferentiated adenocarcinoma while the sarcomatous component was primarily of no special type. A p16 immunostain was performed as a surrogate marker for human papillomavirus (HPV), and it was interpreted as negative.

**Figure 4:**
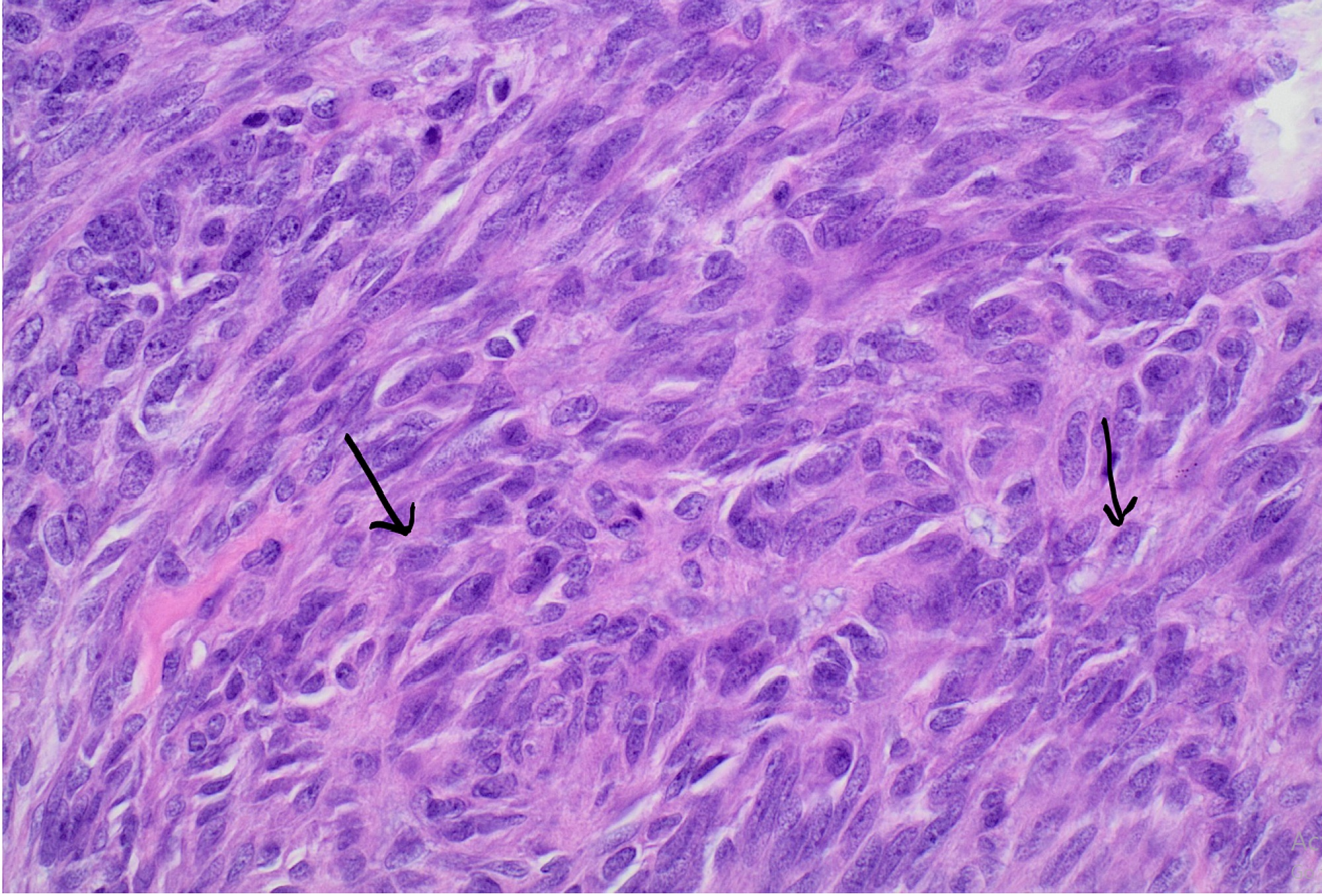
(40X) High magnification of the stromal component

**Figure 5:**
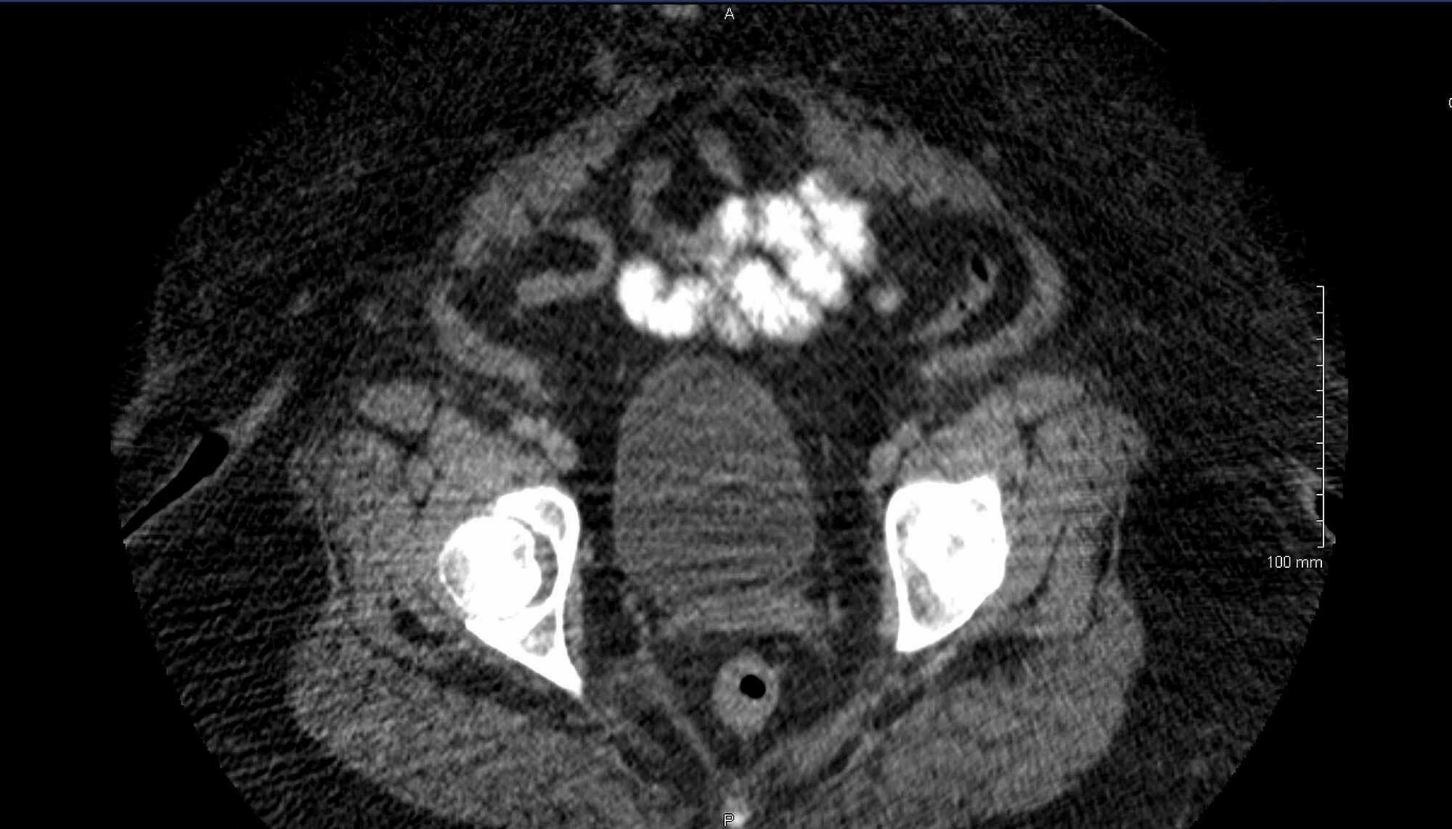
CT pelvis nine months after treatment CT: computed tomography

For further management, she was subsequently transferred to a tertiary care center where she underwent laparotomy with surgical excision of a large calcified right pelvic mass, hysterectomy, omentectomy, and salpingo-oophorectomy. The final pathology report demonstrated an International Federation of Gynecology and Obstetrics (FIGO) Stage IIIb (T3N0M0), poorly differentiated endometrial adenocarcinoma of the uterus with extensive spindle cell proliferation suggesting a sarcomatous component with myometrial invasion greater than 50% and involvement of the cervix (Figure 4). Next-generation sequencing panel analysis of 34 genes associated with hereditary cancer, including BRCA1, BRCA2, MLH1, and MSH6, was negative. She was started on adjuvant chemotherapy consisting of carboplatin and paclitaxel; paclitaxel was changed to nab-paclitaxel during therapy due to the adverse reaction that manifested as shortness of breath, hypotension, and chest pain. Symptoms were reversed with intramuscular epinephrine, intravenous (IV) antihistamines, and glucocorticoids. She completed six cycles of adjuvant chemotherapy. Post-treatment abdominal CT showed no evidence of disease (Figure 5) and CA125 fell to 10 units/ml (1.5-35). Nine months after treatment, the patient's CT abdomen and pelvis showed no evidence of tumor recurrence or metastatic lesions. There was no evidence of recurrence based on a flat CA 125 levels curve. She continues to follow with her local oncologist for surveillance.

## Discussion

Carcinosarcoma of the uterus (UCS), previously called malignant mixed Müllerian tumor (MMMT) is a rare, typically aggressive neoplasm that accounts for less than 5% of all uterine malignancies [4]. In the United States, the incidence is one to four per 100,000 women [5]. It is usually a disease of older postmenopausal women; the median age at diagnosis is 62 to 67 years [4]. Black women have about twice the risk compared to other races/ethnicities [2,5]. Risk factors include obesity, nulliparity, and the use of exogenous estrogen, tamoxifen, and exposure to pelvic radiation are specifically linked to increased risk of developing carcinosarcoma [3]. Hyperinsulinemia is associated with two-fold increased cancer risk, and obesity leads to hyperinsulinemia [6]. Proinflammatory cytokines related to obesity are associated with an increased risk of endometrial carcinomas [7].

Carcinosarcoma arises from a single malignant epithelial clone and is considered to be a high-risk, undifferentiated (metaplastic) variant of endometrial adenocarcinoma. Recent studies showed that carcinomatous and sarcomatous elements are likely derived from a common precursor [5] having mutations that are typical of carcinomas [8].

Vaginal bleeding is the most common presenting sign for USC [9]. Carcinosarcoma is usually hyperechoic compared to the myometrium on ultrasound [10]. CA 125 is the tumor marker being used for follow-up. It correlates with tumor bulk and is used to monitor response and assess the likelihood of metastases and recurrence [11]. Patients should undergo postoperative imaging and re-evaluation of CA 125 to rule out residual or metastatic disease [11]. Uterine carcinosarcoma is surgically staged according to the tumor, node, metastasis (TNM) classification or the International Federation of Gynecology and Obstetrics (FIGO) system. The primary management of uterine carcinosarcoma is surgery for both staging and initial treatment. For patients with stage 4 disease, surgery is of palliative intent. The choice of adjuvant therapy protocol varies based on the stage of the disease [12]. Although the rarity of this neoplasm has precluded larger trials for evaluation for standardized treatment guidelines, Carboplatin and paclitaxel is usually the recommended initial regimen [13]. Some studies suggest combining vaginal brachytherapy (VBT) and chemotherapy. A survival advantage has been demonstrated in women with stage III endometrial cancer treated with chemoradiation (followed by systemic chemotherapy) compared to pelvic radiation therapy alone [14].

Prognosis is usually poor [4]. The stage is the most important prognostic factor. Negative prognostic factors include elevated CA 125, black race, lymphovascular space invasion, myometrial invasion, and the presence of gross residual disease [5,11].

## Conclusions

Uterine carcinosarcoma is a rare, aggressive malignancy that is more common in older black females. It carries a worse prognosis than other endometrial carcinomas. Our patient had an unusual presentation of being a young, Caucasian woman, but she was morbidly obese. Hyperinsulinemia is associated with two-fold increased cancer risk, and obesity is known to lead to hyperinsulinemia. Given the aggressive nature of the disease, early detection and correct diagnosis via rigorous histopathological evaluation, as well as choosing the correct treatment approach, are of extreme clinical importance.
